# Left ventricular concentric remodeling in normal aging is associated with decline of diastolic function assessed by multi-modality imaging

**DOI:** 10.1186/1532-429X-13-S1-P351

**Published:** 2011-02-02

**Authors:** Danielle Janosevic, Kathleen Bertman, Marguerite Roth, William Schapiro, Michael Passick, Simcha Pollack, Nathaniel Reichek, Jie Jane Cao

**Affiliations:** 1 St. Francis Hospital, Roslyn, NY, USA

## Objective

We investigated age related remodeling and associated changes in systolic and diastolic function and myocardial mechanics using cardiac MRI and echocardiography.

## Background

Aging is associated with left ventricular (LV) remodeling. However, relationships between age related remodeling, changes in systolic and diastolic function and changes in myocardial mechanics are not well characterized.

## Methods

Sixty-five normal volunteers aged 23-85 years were carefully screened by history, ECG and echocardiography. All were free of obesity, diabetes, hypertension, cardiovascular and valvular disease. Participants underwent cardiac MRI and echocardiography on the same day. LV structure and global systolic function were assessed using SSFP cine imaging. LV systolic and diastolic torsion and strain were assessed from tagged gradient echo cines using HARP software. Diastolic function was also assessed using echocardiographic mitral inflow and tissue velocity.

## Results

Mean age was 53.6 ± 14 years, 52% female. The cohort was divided into 3 subgroups by age: ≤ 44, 45-60 and ≥ 61 years. Comparing younger to older age groups, LVEDV decreased with aging (r=-0.50, p<0.01) with unchanged LV mass (r=-0.14, p=NS), resulting in an increased mass/volume ratio (0.58 ± 0.1 g/ml, 0.63 ± 0.1 g/ml, 0.68 ± 0.1 g/ml, p=0.01) consistent with concentric remodeling. Torsion increased significantly in the oldest group (1.41 ± 0.4^o^/cm, 1.31 ± 0.4 ^o^/cm, 1.83 ± 0.6 ^o^/cm, p=0.02) while LV global systolic function was unchanged (LVEF 57.9 ± 4.7%, 57.3 ± 4.5%, 60.2 ± 4.5%, p=0.13) and circumferential strain tended to decrease with aging (-19.6 ± 2.5 %, -18.8 ± 1.6 %, -18.1 ± 2.5 %, p=0.06). Diastolic function was consistently reduced with aging evident by decreased mitral medial e’ (10.7 ± 2.0 cm/s, 8.7 ± 1.9 cm/s, 7.0 ± 1.4 cm/s, p<0.01) decreased mitral inflow E/A ratio (1.52 ± 0.46, 1.2 ± 0.35, 1.0 ± 0.2, p<0.01) but increased mitral E/e’ (7.39 ± 1.4, 8.44 ± 2.5, 10.2 ± 2.6, p<0.01). There was also a significant decrease in early diastolic strain rate (54.3 ± 18 %/s, 44.03 ± 13.4 %/s, 35.6 ± 24.6 %/s, p<0.01). Women demonstrated greater declines in diastolic function than men.

## Summary

In this carefully screened normal cohort, age dependent concentric remodeling was associated with significant decline in diastolic global function and mechanics, increased systolic torsion with preserved LVEF and a mild trend toward reduced systolic strain. Age related changes in diastolic function were more pronounced in women than in men.

